# Association between diabetes mellitus and active tuberculosis in Africa and the effect of HIV

**DOI:** 10.1111/tmi.12822

**Published:** 2017-01-09

**Authors:** S. L. Bailey, H. Ayles

**Affiliations:** ^1^ LSHTM TB Centre and Department of Clinical Research London School of Hygiene and Tropical Medicine London UK; ^2^ Zambart Lusaka Zambia

**Keywords:** Diabetes mellitus, tuberculosis, HIV, Africa, systematic review

## Abstract

**Objective:**

To determine current evidence for the association between diabetes and active tuberculosis in Africa, and how HIV modifies, or not, any association between diabetes and active tuberculosis.

**Methods:**

We conducted a systematic review by searching the EMBASE, Global Health and MEDLINE databases. Studies were eligible for inclusion if they explored the association between diabetes mellitus prevalence and active tuberculosis incidence or prevalence, used a comparison group, were conducted in an African population and adjusted the analysis for at least age. Study characteristics were compared, and risk of bias was assessed. The range of effect estimates was determined for the primary association and for effect modification by HIV.

**Results:**

Three eligible studies were identified: two investigated the primary association and two investigated HIV as a potential effect modifier. All studies were case–control studies, including a combined total of 1958 tuberculosis cases and 2111 non‐tuberculosis controls. Diabetes diagnostic methods and analysis strategies varied between studies. Individual study adjusted odds ratios of active tuberculosis for the effect of diabetes mellitus (unstratified) ranged from 0.88 (95% CI 0.17–4.58) to 10.7 (95% CI 4.5–26.0). Individual study *P*‐values for HIV interaction ranged from 0.01 to 0.83. Quantitative synthesis of individual study data was not performed due to heterogeneity between studies.

**Conclusions:**

Few data currently exist on the association between diabetes and active tuberculosis in Africa, and on the effect of HIV on this association. Existing data are disparate. More regional research is needed to guide policy and practice on the care and control of tuberculosis and diabetes in Africa.

## Abstract (fr)

**Objectif:**

Déterminer les preuves actuelles de l'association entre le diabète et la tuberculose active en Afrique et comment le VIH modifie ou non toute association entre le diabète et la tuberculose active.

**Méthodes:**

Nous avons effectué une revue systématique en recherchant dans les bases de données EMBASE, Global Health et Medline. Les études étaient admissibles à l'inclusion si elles ont exploré l'association entre la prévalence du diabète sucré et l'incidence ou la prévalence de la tuberculose active, ont utilisé un groupe de comparaison, ont été effectuées dans une population africaine et ont effectué un ajustement dans l'analyse pour au moins l’âge. Les caractéristiques des études ont été comparées et le risque de biais a été évalué. La gamme d'estimations de l'effet a été déterminée pour l'association primaire et pour la modification de l'effet par le VIH.

**Résultats:**

Trois études admissibles ont été identifiées: deux ont investigué l'association primaire et deux ont investigué le VIH comme un modificateur potentiel de l'effet. Toutes les études étaient des études cas‐témoins, comprenant un total combiné de 1958 cas de tuberculose et 2111 témoins non tuberculeux. Les méthodes de diagnostic du diabète et les stratégies d'analyse variaient d'une étude à l'autre. Les odds ratios (rapports de cotes) ajustés de la tuberculose active pour l'effet du diabète sucré (non stratifié) dans chaque étude allaient de 0,88 (IC95%: 0,17‐4,58) à 10,7 (IC95%: 4,5‐26,0). Les valeurs p de chaque étude pour l'interaction avec le VIH variaient de 0,01 à 0,83. La synthèse quantitative des données de chaque étude n'a pas été réalisée en raison de l'hétérogénéité entre les études.

**Conclusions:**

Peu de données existent actuellement sur l'association entre le diabète et la tuberculose active en Afrique, et sur l'effet du VIH sur cette association. Les données existantes sont disparates. Davantage de recherches régionales sont nécessaires pour orienter les politiques et les pratiques sur les soins et le contrôle de la tuberculose et du diabète en Afrique.

## Abstract (es)

**Objetivo:**

Determinar la evidencia que existe actualmente sobre la asociación entre diabetes y tuberculosis activa en África, y como el VIH modifica o no dicha asociación.

**Métodos:**

Hemos llevado a cabo una revisión sistemática utilizando las bases de datos de EMBASE, Global Health y Medline. Los estudios eran elegibles para ser incluidos si exploraban la asociación entre la prevalencia de diabetes mellitus y la incidencia o prevalencia de tuberculosis activa, tenían un grupo de comparación, se habían llevado a cabo entre una población Africana y el análisis se había ajustado para al menos un grupo de edad. Las características del estudio se compararon y se evaluó el riesgo de sesgo. Se determinó el rango de las estimaciones del efecto para la asociación primaria y el efecto de modificación por VIH.

**Resultados:**

Se identificaron tres estudios elegibles: dos investigaban la asociación primaria y dos investigaban el VIH como un modificador potencial del efecto. Todos eran estudios caso‐control, incluyendo un total combinado de 1,958 casos de tuberculosis y 2,111 controles no‐tuberculosos. Los métodos de diagnóstico de diabetes y las estrategias de análisis variaban entre estudios. La razón de probabilidades ajustada de la tuberculosis activa sobre el efecto sobre la diabetes mellitus (sin estratificar) y para un estudio individual estaba entre 0.88 (IC 95% 0.17‐4.58) y 10.7 (IC 95% 4.5‐26.0). Los valores p para la interacción con VIH de los estudios individuales estaban en un rango de 0.01 a 0.83. La síntesis cuantitativa de los datos individuales de los estudios no se llevó a cabo debido a la heterogeneidad entre estudios.

**Conclusiones:**

Actualmente existen pocos datos sobre la asociación entre diabetes y tuberculosis activa en África, y sobre el efecto del VIH en esta asociación. Los datos existentes son dispares. Se requiere más investigación a nivel regional para informar las políticas y prácticas en la atención y el control de la tuberculosis y diabetes en África.

## Introduction

An association between diabetes mellitus (DM) and active tuberculosis (TB) has been established: systematic reviews and meta‐analyses of studies exploring the relationship suggest that the incidence of active TB is two to three times higher in those with DM than those without DM 1, 2, 3. However, the data contributing to this body of evidence originate almost entirely from countries outside of Africa 1, 2, 3. There is longitudinal evidence that suggests this association may differ in African populations. A Danish study evaluated the effect of ethnicity and DM on the risk of incident TB over a follow‐up period of 15 years through linking nationwide DM and TB registers at case level 4. They found a TB rate ratio of 1.9 in individuals with DM *vs*. individuals without DM, regardless of country of birth, with the exception of African‐born individuals who had a rate ratio of 0.5. An ecological longitudinal study covering the years 2000 and 2012 studied the global relationship between the prevalence of DM and the incidence of TB to evaluate their coexistence worldwide 5. Only countries with a high DM prevalence (>7.6%) showed a significant positive association between DM prevalence and TB incidence based on linear regression time trend analysis (*r* = 0.17, *P* = 0.013). A non‐significant inverse relationship was found for the African region (*r* = −0.27).

Whilst there is no reason to suspect the underlying pathophysiology of the association should differ between ethnographic populations, the context of the association in Africa could be different from the rest of the world and consequently lead to a different overall association. Other risk factors for tuberculosis could act as effect modifiers on the association. The most notable difference relating to tuberculosis risk factors between Africa and elsewhere is the high prevalence of HIV in Africa, which is more than five times higher than in any other world region 6.

The dual effect of diabetes and HIV on the risk of developing TB disease or on its clinical evolution is unclear. It could be that the effect of hyperglycaemia on TB risk is relatively small in HIV‐positive individuals compared with its effect in HIV‐negative individuals, as the greatly increased risk of TB among HIV‐positive individuals could diminish any additional increased risk from hyperglycaemia. On the other hand, the effect of hyperglycaemia might be exacerbated in the presence of HIV infection if the contributions of each to increased TB risk are synergistic.

The prevalence and incidence of tuberculosis remain high in many parts of Africa 7. The number of adults with diabetes in Africa is predicted to rise from 14.2 million in 2015 (uncertainty interval 9.5–29.4 million) to 34.2 million in 2040 (uncertainty interval 23.7–67.7 million) 8, 9, 10, 11. To appropriately respond to this now and prepare for a future higher prevalence of diabetes, a deeper understanding of associations between diabetes and tuberculosis in the context of Africa is needed. Therefore, we aimed to undertake systematic reviews to determine current evidence for, firstly, the association between the prevalence of diabetes and the incidence or prevalence of active tuberculosis in Africa and, secondly, how HIV modifies, or not, any association between the prevalence of diabetes and the incidence or prevalence of tuberculosis.

## Methods

The EMBASE, Global Health and PubMed databases were searched. Studies that investigated the relationship between the prevalence of diabetes and the incidence or prevalence of active tuberculosis, included a comparison group, were conducted in an African population and adjusted for age were eligible for inclusion. As age has the potential to be a major confounder of the association, studies that did not adjust for at least age in the analysis, or present data that enabled this analysis, were not eligible. Reference lists of identified eligible papers were additionally hand‐searched to identify further potentially relevant studies.

The EMBASE Classic+EMBASE database was searched for publications from 1947 until June 2016, the Global Health database was searched for publications from 1910 until June 2016, and the Ovid MEDLINE^®^ database was searched for publications from 1946 until June 2016, including Epub Ahead of Print, In‐Process, Other Non‐Indexed Citations and Ovid MEDLINE^®^ Daily. The search terms and strategies used were deliberately broad to increase the likelihood of all relevant studies being identified. Searches were restricted to human studies. No restriction on language was made. The following MESH and text search terms were used as follows:

- Diabetes mellitus.mp. [mp = title, abstract, heading word, original title, keyword]
- Hyperglycaemia.mp. [mp = title, abstract, heading word, original title, keyword]
- 1 OR 2
- Tuberculosis.mp. [mp = title, abstract, heading word, original title, keyword]
- Africa.mp. [mp = title, abstract, heading word, original title, keyword]
- 3 AND 4 AND 5

Deduplication of papers identified by the searches was performed using the Ovid database platform. The titles and abstracts were screened for eligibility, and the full texts of articles identified as potentially relevant were examined. All studies meeting the eligibility criteria were included for exploration of the first aim, to determine the association between the prevalence of diabetes and the incidence or prevalence of active tuberculosis in Africa. Studies that met the eligibility criteria and presented the primary age‐adjusted association stratified by HIV were also included in assessment of the second aim, to determine how HIV modifies, or not, any association between the prevalence of diabetes and the incidence or prevalence of tuberculosis.

It is possible that studies relevant to the second aim could take place in non‐African populations, and so to ensure all relevant papers were identified, a second search was performed using the same databases and the same search strategy, except no restriction on location was made and the following MESH and text search terms were used: Diabetes mellitus OR hyperglycaemia AND tuberculosis AND HIV. Studies were eligible only if they investigated the primary association between DM prevalence and TB incidence/prevalence and stratified the analysis by HIV. Studies that investigated the prevalence of diabetes among TB patients stratified by HIV but did not include a non‐TB population were not eligible for inclusion because it is not possible to assess for effect modification without data from a control population for the primary association.

Individual study data were extracted from reports using data collection tables, to identify individual study characteristics, risk of bias and results. When necessary, study authors were contacted for clarification of study data. Study characteristics sought were period of data collection, study design, study setting, study size and HIV prevalence among study participants. The risk of bias for individual studies was assessed by ascertaining study definitions for the exposure variable, outcome variable and comparison group and determining the variables adjusted for in the analysis. Qualitative description was used to synthesise the risk of bias results. The principal summary measures sought were the odds ratios or risk ratios for the association between DM and TB, overall and stratified by HIV. The range of effect estimates was determined for the primary association and stratified by HIV. The range of *P*‐values for interaction was also determined.

The study protocol was not eligible for registration in current prospective registers of systematic reviews protocol because the review does not investigate an intervention or strategy to prevent, diagnose, treat or monitor a health condition; rather, it investigates observational associations.

## Results

**Figure 1:**
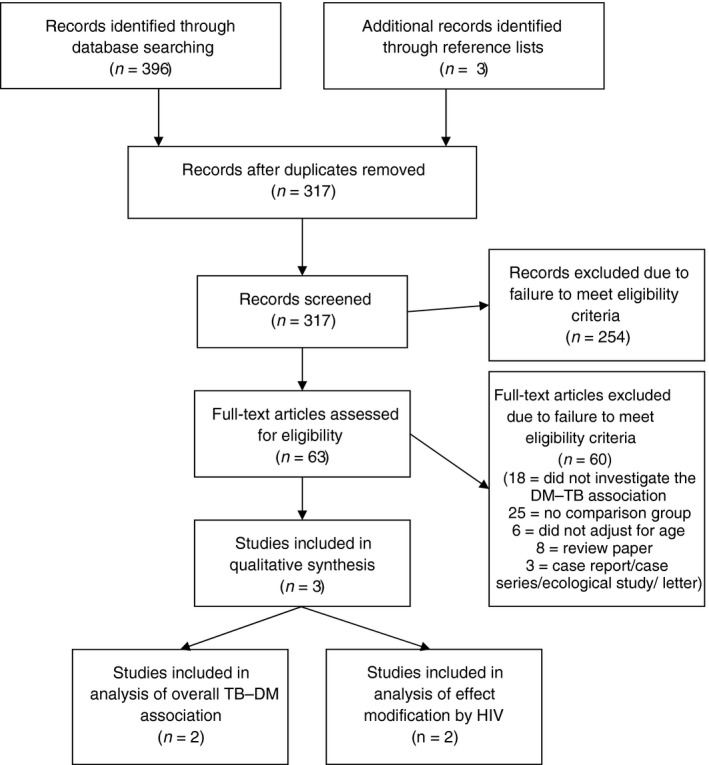
Flow diagram of study selection for papers investigating the association between diabetes mellitus prevalence and tuberculosis incidence or prevalence in an African population.

The database searches for studies investigating the primary association in Africa identified 314 potential papers after deduplication (Figure 1). A further three potentially relevant papers were identified from reference lists. Therefore, 317 titles and abstracts were screened, of which 254 were excluded due to failure to meet the eligibility criteria. We examined 63 full texts, of which three studies were found to meet the eligibility criteria for inclusion. All three papers investigated the association between DM prevalence and TB incidence in an African population 12, 13, 14, although only two reported the overall adjusted effect estimate 13, 14. Of the three identified papers, two studies went on to also investigate HIV as an effect modifier of the association 12, 14. No papers investigating the association between DM prevalence and TB prevalence were identified. The additional database searches for any further papers investigating the primary association plus HIV as an effect modifier identified 800 potentially relevant papers after deduplication, but no further eligible papers were found after screening and full‐text assessment for eligibility.

The database searches identified eight review papers that had relevance to associations between TB, diabetes and HIV 15, 16, 17, 18, 19, 20, 21, 22, although none reported current evidence for the association between DM and TB in Africa and none explored how HIV modifies or not this association. Rather, they focused on related factors including current understanding of the underlying mechanisms of diabetes‐related and HIV‐related increased susceptibility to TB 15, 16, 17, the importance of and challenges faced with TB and diabetes comanagement and control 16, 18, 19, 20, current research gaps and prioritised areas for research relating to TB and diabetes 21, and evidence of association between TB and diabetes from elsewhere in the world 16, 17, 22. None of the review papers identified additional primary research papers that had not already been identified through the database searches.

Individual study characteristics are shown in Table 1. All three studies used a case–control design, investigating a combined total of 1958 tuberculosis cases and 2111 non‐tuberculosis controls. The prevalence of HIV ranged from 23% to 43% among cases and from 10% to 14% among controls.

**Table 1:** : Individual study characteristics and risk of bias

Study	Date of data collection	Region, Country	Study design	Study size	Exposure variable	Outcome variable	Primary comparison	HIV prevalence	Variables adjusted for in analysis
Faurholt‐Jepsen *et al.,* 2011^12^	April 2006 – January 2009	Mwanza, Tanzania	Case–control	803 cases and 350 controls	DM, determined by FCG > 6 mmol/l or 2hCG > 11 mmol/l, measured in cases a few days after initiation of TB treatment	Active pulmonary TB, determined by culture or sputum smear	Newly diagnosed adult TB cases presenting in one of four health facilities and non‐TB age‐ and sex‐matched neighbourhood controls	43.2% among cases, 10.0% among controls; determined using two rapid tests and, if equivocal, ELISA	Age, sex, religion, marital status, occupation in model 1; the above plus the acute phase reactant alpha‐1‐acid glycoprotein in model 2
Haraldsdottir *et al.,* 2015^13^	July 2010 ‐ July 2011	Bissau, Guinea‐Bissau	Case–control	110 cases and 572 controls	DM, determined by FCG ≥ 7.0 mmol/l. measured in cases when they were newly diagnosed with TB	Active pulmonary TB, determined by sputum smear or chest radiograph plus signs and symptoms suggestive of TB after ineffective antibiotic treatment	Newly diagnosed adult TB cases registered by notification system and non‐TB unmatched adult community controls randomly selected from a demographic surveillance database	22.6% among cases, determined using two rapid tests; HIV status not determined for control participants	Age, sex, body mass index
Boillat‐Blanco *et al.,* 2016^14^	July 2012 – June 2014	Kinondoni District, Tanzania	Case–control	539 cases and 496 controls	DM, determined by FCG ≥ 7.0 mmol/L, or 2hCG ≥ 11.1 mmol/l or HbA1c ≥ 6.5%, measured in cases at enrolment and confirmed by repeat testing 2‐5 days later; then repeated after a median of 5 months of antituberculosis treatment	Active TB diagnosed by the National TB and Leprosy Control Programme	Consecutive adults with new active tuberculosis presenting in participating hospitals and sex‐ and age‐matched non‐TB controls selected from adults accompanying patients to the outpatient departments	32% among cases, 14% among controls; determined using two rapid tests	Age, sex, body mass index, socioeconomic status, HIV status (non‐stratified models only)

TB, tuberculosis; DM, diabetes mellitus; FCG, fasting capillary blood glucose; 2hCG, 2‐h capillary blood glucose after a standard 75 g oral glucose tolerance test.

Individual study risk of bias is also shown in Table 1. Definitions of DM and TB varied between each study. All studies tested for DM in TB cases around the time of TB treatment initiation or in newly diagnosed patients with TB. Boillat‐Blanco *et al*. 14 additionally undertook repeat testing for diabetes 5 months after TB treatment initiation. The analysis strategy varied between each study. All adjusted for age and sex. Faurholt‐Jepsen *et al*. presented the results for two separate analysis strategies, one including adjustment for the acute phase reactant alpha‐1‐acid glycoprotein and one without.

**Figure 2:**
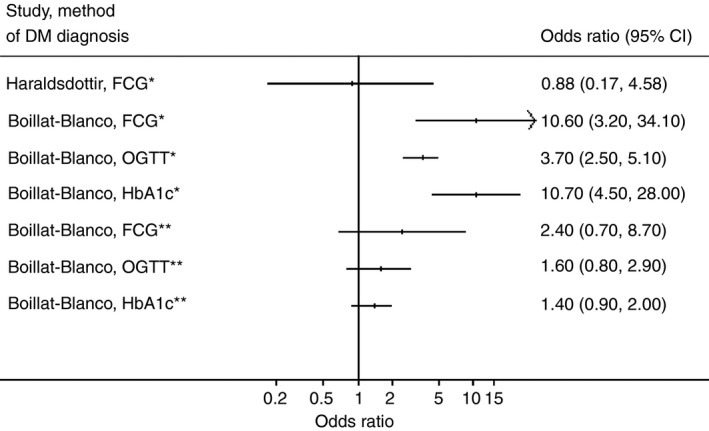
Forest plot for the adjusted odds ratios of active tuberculosis comparing those with diabetes mellitus to those without. DM, diabetes mellitus; FCG, fasting capillary blood glucose; OGTT, oral glucose tolerance test; HbA1c, glycated haemoglobin; *DM in cases measured around the time of TB treatment initiation; **DM in cases measured a median of 5 months after TB treatment initiation.

Table 2 presents the individual study results based on the diabetes tests performed in newly diagnosed TB cases around the time of enrolment. Adjusted odds ratios of TB for the effect of DM range from 0.88 (95% CI 0.17–4.58) to 10.7 (95% CI 4.5–26.0). Figure 2 shows this graphically, along with odds ratios of TB for the effect of DM measured at follow‐up. Boillat‐Blanco *et al*. found the prevalence of DM in cases reverted to the background prevalence of DM in controls after treatment for tuberculosis. Adjusted odds ratios of TB for the effect of DM correspondingly reverted to the null.

**Table 2:** Individual study estimates of the unadjusted and adjusted odds ratios of active tuberculosis comparing individuals with diabetes mellitus to those without, overall and stratified by HIV, with diabetes mellitus measured around the time of TB diagnosis or initiation of TB treatment

Study	Overall					HIV uninfected				HIV infected				*P*‐value for HIV interaction (adjusted analysis)
	Method of DM diagnosis	Number (%) of cases with DM	Number (%) of controls with DM	Unadjusted OR of active TB (95% CI)	Adjusted OR of active TB (95% CI)	Number (%) of cases with DM	Number (%) of controls with DM	Unadjusted OR of active TB (95% CI)	Adjusted OR of active TB (95% CI)	Number (%) of cases with DM	Number (%) of controls with DM	Unadjusted OR of active TB (95% CI)	Adjusted OR of active TB (95% CI)	
Faurholt‐Jepsen D *et al.,* 2011^12^	FCG and OGTT combined	134 (16.7)	33 (9.4)	2.2 (1.5–3.4)	NR	NR	NR	2.15 (1.35–3.42)	Model 1: 2.14 (1.32–3.46)	NR	NR	1.94 (0.65–5.75)	Model 1: 2.05 (0.68–6.19)	Model 1: NR
									Model 2: 4.23 (1.54–11.57)				Model 2: 0.14 (0.01–1.81)	Model 2: 0.01
Haraldsdottir T.L. *et al.,* 2015^13^	FCG	3 (2.8)	11 (2.1)	NR	0.88 (0.17–4.58)	NR	NR	NR	NR	NR	NR	NR	NR	NR
Boillat‐Blanco N *et al.,* 2016^a^ ^14^	FCG (enrolment)	24 (4.5)	6 (1.2)	4.2 (1.7–10.3)	10.6 (3.2–34.1)	15 (4.2)	4 (1.0)	4.9 (1.6–15.0)	8.8 (2.1–36.6)	8 (4.8)	2 (3.0)	1.9 (0.4–9.1)	17.1 (1.6–179.4)	0.83
	OGTT (enrolment)	36 (6.8)	15 (3.1)	2.9 (1.6–5.4)	3.7 (2.5–5.1)	23 (6.5)	10 (2.4)	3.5 (1.6–7.4)	3.8 (1.4–10.5)	12 (7.2)	5 (7.6)	1.4 (0.5–4.0)	3.8 (1.0–15.3)	0.73
	HbA1c (enrolment)	49 (9.3)	11 (2.2)	6.5 (3.3–12.9)	10.7 (4.5–26.0)	33 (9.3)	5 (1.2)	11.8 (4.5–31.0)	19.3 (6.1–61.0)	16 (9.6)	6 (9.1)	1.9 (0.7–5.3)	4.7 (1.1–20.8)	0.048

TB, tuberculosis; OR, odds ratio; CI, confidence interval; OGTT, oral glucose tolerance test; NR, not reported; Model 1: adjusted for age, sex, religion, marital status and occupation; Model 2: adjusted for age, sex, religion, marital status, occupation and serum alpha‐1‐acid glycoprotein.

a Results presented are for initial DM prevalence measured at the time of recruitment.

The individual study effects of HIV on the association between DM and TB were different depending on the definition of diabetes and the factors adjusted for in the analysis (Table 2). Boillat‐Blanco *et al*. found no evidence for effect modification by HIV other than when diabetes was determined by HbA1c and measured at the time of enrolment, in which case individuals who were uninfected with HIV had a stronger association between DM and TB than individuals infected with HIV (*P* = 0.048). Faurholt‐Jepsen *et al*. also found evidence for a stronger association among HIV uninfected individuals but only when adjusting for alpha‐1‐acid glycoprotein (*P* = 0.01). Quantitative synthesis of individual study data was not performed due to heterogeneity between studies.

## Discussion

This systematic review identified only three eligible studies conducted in African populations. Only two of these studies presented data on the adjusted association between DM and TB. One found no evidence of association and the other found evidence of a positive association only when diabetes was measured in cases around the time of TB treatment initiation. There was no evidence of association when DM was measured in TB cases after receiving TB treatment, suggesting that the initial association seen was due to an increase in stress‐induced hyperglycaemia among newly diagnosed TB cases rather than due to true diabetes.

Only two studies stratified their analysis by HIV. The results depended on the analysis strategy and method of DM diagnosis used. There was little evidence of association among either HIV‐infected or uninfected individuals when DM was measured in cases after TB treatment.

The results seen in this systematic review are in keeping with the idea that the association between DM and active TB may be different among African populations, but existing data are currently too sparse to be conclusive. It remains possible that HIV could modify the association, but again, there are currently insufficient data to be certain.

A limitation at the individual study level was the different criteria used for diabetes diagnosis in each study, both for the methods and the glycaemic cut‐offs used to determine diabetes. This made comparison between studies problematic. Conformity with WHO diagnostic criteria and presenting separate rather than combined analyses for each method of diagnosis used would mitigate this limitation.

The results of this systematic review, and any future repeat review, have relevance to healthcare policymakers, academics and practitioners in Africa. The global collaborative framework for the care and control of tuberculosis and diabetes produced in 2011 by the World Health Organization and the International Union Against Tuberculosis and Lung Disease had few contributory studies from Africa 23. In 2016, this systematic review suggests there remain few studies based in African populations that can guide local and regional policy and practice on the care and control of TB and diabetes. It remains possible that the association between diabetes and active TB could be different in African populations from elsewhere in the world, and it remains possible that HIV could modify the association. Given the continued high incidence of tuberculosis in much of Africa and the predicted rising prevalence of diabetes throughout Africa 7, 8, further evidence on the nature and magnitude of their association in this setting would be valuable.

## Conclusions

Few data currently exist on the association between diabetes and active tuberculosis in Africa, or on the effect of HIV on this association. Exploration of diabetes diagnosed both at the time of TB diagnosis and after TB treatment is valuable to distinguish between diabetes and stress‐induced hyperglycaemia secondary to infection with TB. More regional research is needed to guide policy and practice on the care and control of tuberculosis and diabetes in Africa.
